# Proteogenomic and observational evidence implicate ANGPTL4 as a potential therapeutic target for colorectal cancer prevention

**DOI:** 10.1093/jnci/djaf137

**Published:** 2025-06-13

**Authors:** James Yarmolinsky, Matthew A Lee, Evelyn Lau, Ferran Moratalla-Navarro, Emma E Vincent, Ruifang Li-Gao, Patrick C N Rensen, Ko Willems van Dijk, Kostas K Tsilidis, Apiwat Sangphukieo, Elmira Ebrahimi, Jochen Hampe, Loïc Le Marchand, Franzel J B van Duijnhoven, Kala Visvanathan, Michael O Woods, Marcela Guevara, Sabina Sieri, Giovanna Masala, Keren Papier, Shama Virani, Tom Dudding, Abbas Dehghan, Alexander G Smith, Dennis Wang, Victor Moreno, Marc J Gunter, Ioanna Tzoulaki

**Affiliations:** Department of Epidemiology and Biostatistics, School of Public Health, Imperial College London, London, United Kingdom; Nutrition and Metabolism Branch (NME), International Agency for Research on Cancer, Lyon, France; Institute for Human Development and Potential, Agency for Science, Technology and Research (A*STAR), Singapore, Republic of Singapore; Unit of Biomarkers and Susceptibility (UBS), Oncology Data Analytics Program (ODAP), Catalan Institute of Oncology (ICO), L'Hospitalet del Llobregat, Barcelona, Spain; ONCOBELL Program, Bellvitge Biomedical Research Institute (IDIBELL), L'Hospitalet de Llobregat, Barcelona, Spain; Consortium for Biomedical Research in Epidemiology and Public Health (CIBERESP), Madrid, Spain; Department of Clinical Sciences and Universitat de Barcelona Institute of Complex Systems (UBICS), Faculty of Medicine, University of Barcelona, Barcelona, Spain; MRC Integrative Epidemiology Unit, University of Bristol, Bristol, United Kingdom; Population Health Sciences, Bristol Medical School, University of Bristol, Bristol, United Kingdom; Translational Health Sciences, Bristol Medical School, University of Bristol, Bristol, United Kingdom; Department of Clinical Epidemiology, Leiden University Medical Center, Leiden, The Netherlands; Division of Endocrinology, Department of Internal Medicine, Leiden University Medical Center, Leiden, The Netherlands; Einthoven Laboratory for Experimental Vascular Medicine, Leiden University Medical Center, Leiden, The Netherlands; Division of Endocrinology, Department of Internal Medicine, Leiden University Medical Center, Leiden, The Netherlands; Einthoven Laboratory for Experimental Vascular Medicine, Leiden University Medical Center, Leiden, The Netherlands; Department of Human Genetics, Leiden University Medical Center, Leiden, The Netherlands; Department of Epidemiology and Biostatistics, School of Public Health, Imperial College London, London, United Kingdom; Department of Hygiene and Epidemiology, University of Ioannina School of Medicine, Ioannina, Greece; Genomic Epidemiology Branch, International Agency for Research on Cancer, Lyon, France; Genomic Epidemiology Branch, International Agency for Research on Cancer, Lyon, France; Department of Medicine I, University Hospital Dresden, Technische Universität Dresden (TU Dresden), Dresden, Germany; Epidemiology Program, University of Hawaii Cancer Center, Honolulu, HI, United States; Division of Human Nutrition and Health, Wageningen University & Research, Wageningen, The Netherlands; Department of Epidemiology, Johns Hopkins Bloomberg School of Public Health, Baltimore, MD, United States; Discipline of Genetics, Memorial University of Newfoundland, St John's, NL, Canada; Instituto de Salud Pública y Laboral de Navarra, Pamplona, Spain; Centro de Investigación Biomédica en Red de Epidemiología y Salud Pública (CIBERESP), Madrid, Spain; Navarra Institute for Health Research (IdiSNA), Pamplona, Spain; Epidemiology and Prevention Unit, Fondazione IRCCS Istituto Nazionale dei Tumori, Milan, Italy; Clinical Epidemiology Unit, Institute for Cancer Research, Prevention and Clinical Network (ISPRO), Florence, Italy; Cancer Epidemiology Unit, Nuffield Department of Population Health, University of Oxford, Oxford, United Kingdom; Genomic Epidemiology Branch, International Agency for Research on Cancer, Lyon, France; Bristol Dental School, University of Bristol, Bristol, United Kingdom; Department of Epidemiology and Biostatistics, School of Public Health, Imperial College London, London, United Kingdom; Dementia Research Institute, Imperial College London, London, United Kingdom; Department of Epidemiology and Biostatistics, School of Public Health, Imperial College London, London, United Kingdom; Institute for Human Development and Potential, Agency for Science, Technology and Research (A*STAR), Singapore, Republic of Singapore; National Heart and Lung Institute, Imperial College London, London, United Kingdom; Unit of Biomarkers and Susceptibility (UBS), Oncology Data Analytics Program (ODAP), Catalan Institute of Oncology (ICO), L'Hospitalet del Llobregat, Barcelona, Spain; ONCOBELL Program, Bellvitge Biomedical Research Institute (IDIBELL), L'Hospitalet de Llobregat, Barcelona, Spain; Consortium for Biomedical Research in Epidemiology and Public Health (CIBERESP), Madrid, Spain; Department of Clinical Sciences and Universitat de Barcelona Institute of Complex Systems (UBICS), Faculty of Medicine, University of Barcelona, Barcelona, Spain; Department of Epidemiology and Biostatistics, School of Public Health, Imperial College London, London, United Kingdom; Nutrition and Metabolism Branch (NME), International Agency for Research on Cancer, Lyon, France; Department of Epidemiology and Biostatistics, School of Public Health, Imperial College London, London, United Kingdom; Dementia Research Institute, Imperial College London, London, United Kingdom; Systems Biology, Biomedical Research Foundation Academy of Athens, Athens, Greece

## Abstract

**Background:**

The role of lipid-perturbing medications in cancer risk is unclear.

**Methods:**

We employed *cis*-Mendelian randomization and colocalization to evaluate the role of 5 lipid-perturbing drug targets (ANGPTL3, ANGPTL4, APOC3, CETP, and PCSK9) in risk of 5 cancers (breast, colorectal, head and neck, ovarian, and prostate). We triangulated findings using pre-diagnostic protein measures in prospective analyses in EPIC (977 colorectal cancer cases, 4080 sub-cohort members) and the UK Biobank (860 colorectal cancer cases, 50 177 controls). To gain mechanistic insight into the role of ANGPTL4 in carcinogenesis, we examined the impact of the ANGPTL4 p. E40K loss-of-function variant on differential gene expression in normal colon tissue in BarcUVa-Seq. Finally, we evaluated the association of colon tumor *ANGPTL4* expression with cancer-specific mortality in TCGA.

**Results:**

In analysis of 78 473 cases and 107 143 controls, genetically proxied circulating ANGPTL4 inhibition was associated with reduced colorectal cancer risk (OR_SD decrease_ = 0.76, 95% confidence interval [CI] = 0.66 to 0.89, *P *= 5.52 × 10^−4^, PP_colocalization_ = 0.83). This association was replicated using pre-diagnostic circulating ANGPTL4 concentrations in EPIC (hazard ratio [HR]_log10 decrease_ = 0.91, 95% CI = 0.84 to 0.98, *P *= .01) and the UK Biobank (HR_SD decrease_ = 0.93, 95% CI = 0.86 to 0.99, *P *= .03). In gene-set enrichment analysis of differential gene expression in 445 colon tissue samples, ANGPTL4 loss-of-function down-regulated several cancer-related biological pathways (*P*_FDR_ < .05), including those involved in cellular proliferation, epithelial-to-mesenchymal transition, and bile acid metabolism. In analysis of 465 colon cancer patients, lower *ANGPTL4* tumor expression was associated with reduced colorectal cancer-specific mortality risk (HR_log2 decrease_ = 0.66, 95% CI = 0.50 to 0.87, *P *= 2.92 × 10^−3^).

**Conclusions:**

Our integrative proteogenomic and observational analyses suggest a potential protective role of lower circulating ANGPTL4 concentrations in colorectal cancer risk. These findings support further evaluation of ANGPTL4 as a therapeutic target for colorectal cancer prevention.

## Introduction

Lipids perform various essential physiological functions including providing an energy reserve, serving as structural components of cell membranes, and participating in cellular signaling.^1^ It is well established that select lipid parameters (eg, LDL cholesterol) contribute to atherosclerotic cardiovascular disease (ASCVD). Along with their role in ASCVD, preclinical studies have suggested that lipids may also influence carcinogenesis through several mechanisms including those related to insulin resistance, inflammation, and oxidative stress.^2^ Reprogramming of lipid metabolism also plays a critical role in promoting tumorigenesis and is considered an emerging hallmark of cancer.^3^^,^^4^ For example, cancer cells must harness lipid metabolism to support cell division, adapt to stress, and enable metastatic dissemination.^5-7^ In addition, lipid metabolic reprogramming can remodel the tumor microenvironment by influencing the recruitment, survival, and function of immune cells.^8^

Consistent with a role of circulating lipids in cancer development, preclinical and epidemiological studies have suggested that several lipid-perturbing medications may lower cancer risk.^9-13^ For example, knockdown of ANGPTL3, the target of the lipid-lowering therapy evinacumab, has been shown to suppress proliferation, migration, and invasion in several cancer cell lines.^14-16^ In addition, PCSK9 inhibition using siRNA, gene knockout, or anti-PCSK9 vaccination promotes apoptosis in in vitro cancer models.^13^ Observational epidemiological studies have also reported that long-term statin users have lower rates of site-specific cancer as compared with non-users.^12^^,^^17-21^

These findings collectively suggest the potential for repurposing approved and/or emerging lipid-perturbing cardiovascular disease (CVD) medications for cancer prevention. However, in the absence of randomized clinical trial data, the causal nature of these medications in cancer onset, and thus their suitability as intervention targets, is unclear. This is because of the uncertain relevance of preclinical disease models to humans and the susceptibility of conventional observational analyses to residual confounding and reverse causation, undermining confident causal inference.^22-24^

Here, we leveraged 4 complementary epidemiological approaches to triangulate evidence on the potential causal role of lipid-perturbing drug targets in cancer risk. We employed drug-target Mendelian randomization (MR) to systematically evaluate the effect of 5 approved or emerging lipid-perturbing drug targets for CVD (APOC3, ANGPTL3, ANGPTL4, CETP, and PCSK9) on risk of 5 cancers (breast, colorectal, head and neck, ovarian, and prostate). This approach leverages the natural randomization of germline genetic variants at meiosis and can minimize conventional epidemiological issues of confounding and reverse causation. We then examined the association of pre-diagnostic direct measures of circulating protein targets and cancer risk in prospective analyses in the European Prospective Investigation into Cancer and Nutrition (EPIC) and the UK Biobank. To gain mechanistic insight into the role of ANGPTL4 in carcinogenesis, we explored the impact of ANGPTL4 loss-of-function on differential gene expression in normal colon tissue samples in the University of Barcelona and the University of Virginia Genotyping and RNA Sequencing (BarcUVa-Seq) project. Finally, to explore whether ANGPTL4 is involved in cancer progression, we evaluated the association of *ANGPTL4* gene expression in colon tumor tissue with all-cause mortality in The Cancer Genome Atlas (TCGA).

## Methods

**Figure 1.**
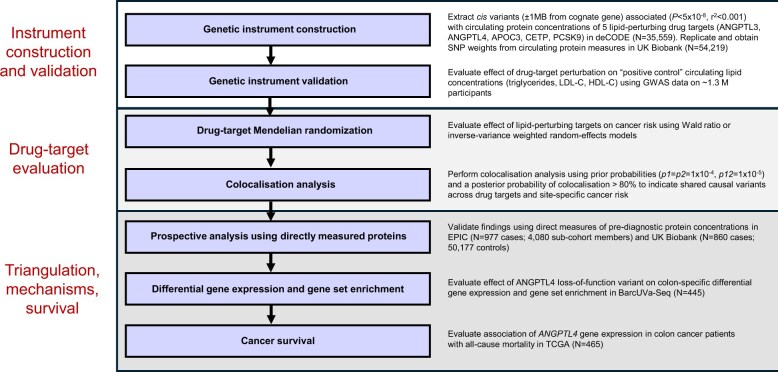
EPIC = European Prospective Investigation into Cancer and Nutrition; GWAS = genome-wide association study; TCGA = The Cancer Genome Atlas.

A step-by-step overview of the analytical stages of this work is presented in Figure 1.

### Study populations

We selected cancer outcomes where there was prior evidence from Mendelian randomization studies to suggest a role of circulating lipids (ie, breast, colorectal) or lipid-perturbing drug targets (ie, head and neck, ovarian, prostate) in their etiology.^25-31^

For drug-target MR analyses, summary genetic association estimates for overall and histological or anatomical subtype-stratified cancer risk were obtained from analyses of up to 319 661 cancer cases and 348 078 controls across 5 consortia.^32-36^ For genetic instrument validation analyses, summary genetic association data on circulating LDL cholesterol, HDL cholesterol, and triglycerides were obtained from analyses of ∼1.3 million participants in the Global Lipids Genetics Consortium (GLGC) analysis.^37^ These analyses were restricted to participants of European ancestry. All studies contributing data to these analyses had the relevant institutional review board approval from each country, in accordance with the Declaration of Helsinki, and all participants provided informed consent.

For observational analyses using direct measures of protein drug targets, we used data from the EPIC cohort study.^38^ We limited analyses to 10 261 individuals recruited into a multi-endpoint case-cohort within EPIC, of whom 6876 had incident cancer, including 977 colorectal cancer cases (658 colon cancer, 319 rectal cancer).

For transcriptomic analyses examining the effect of ANGPTL4 loss-of-function on differential gene expression, we obtained combined germline genotype and RNA sequencing data from 445 normal (ie, non-neoplastic) colon tissue samples in BarcUVa-Seq.^39^

For analyses examining the association of tumor gene expression with all-cause mortality, we obtained gene expression (RNA-Seq), demographic, and clinicopathological data from 465 colon adenocarcinoma (TCGA-COAD) cases in TCGA.^40^

### Genetic instrument construction

Genetic instruments for drug targets were constructed using a 2-stage strategy to minimize bias from “Winner’s curse” (further information on instrument construction is presented in the Supplementary Methods). In brief, genetic instruments were constructed from genome-wide significant (*P *< 5 × 10^−8^) and independent (LD r^2^ < 0.001) single-nucleotide polymorphisms (SNPs) in or within 1MB from the gene encoding the relevant protein (*N* = 35 559 individuals of Icelandic ancestry) then tested for replication in an independent GWAS of 54 219 individuals of primarily white British ancestry in the UK Biobank.^41^^,^^42^ For SNPs that replicated (*P *< .05) and were directionally consistent, SNP weights were obtained from UK Biobank analyses. Genetic instruments for lipid-perturbing drug targets were constructed using circulating plasma protein levels (ie, as compared with downstream biomarkers of drug target perturbation) to increase instrument strength given that circulating plasma protein levels are expected to be more proximal to *cis*-acting variants influencing these traits.

### Drug-target Mendelian randomization primary and sensitivity analyses

Drug-target Mendelian randomization uses germline genetic variants as instrumental variables to estimate on-target effects of perturbation of drug targets, under specific assumptions (described in the Supplementary Methods).

For drug targets instrumented by a single SNP, the Wald ratio was used to generate effect estimates and the delta method was used to approximate standard errors. For drug targets instrumented by 2 or more SNPs, inverse-variance weighted (IVW) random-effects models were used to estimate causal effects.^43^

We tested the “relevance” assumption by generating estimates of the proportion of variance of each drug target explained by the instrument (r^2^) and F-statistics. We evaluated the “exclusion restriction” assumption by performing various sensitivity analyses. First, we validated our instruments by evaluating the effect of genetically proxied drug targets on downstream biomarkers influenced (ie, for approved drugs) or presumed to be influenced (ie, for emerging drugs) by the target as “positive control” analyses (ie, triglyceride concentrations for APOC3, ANGPTL3, ANGPTL4; HDL cholesterol concentrations for CETP; LDL cholesterol concentrations for PCSK9).^25^^,^^44-47^ Second, colocalization was performed to evaluate whether drug targets and both “positive control” lipid measures and cancer outcomes showing evidence of association in MR analyses (Bonferroni-corrected *P *< 6.17 × 10^−4^) were likely to share the same causal variant at a given locus. We used a posterior probability of colocalization (PP_colocalization_) > 0.80 to support colocalization of drug targets and disease outcomes. Third, for analyses examining the association of circulating triglycerides with colorectal cancer risk, we employed 3 complementary “pleiotropy-robust” models: MR-Egger regression, weighted median estimation, and weighted mode estimation.^48-50^

To account for multiple testing across drug-target MR analyses, a Bonferroni correction was used (*P *< 6.17 × 10^−4^) (false-positive rate = 0.05/81 statistical tests).

### Association of pre-diagnostic ANGPTL4 concentrations and colorectal cancer risk

In analysis of directly measured pre-diagnostic ANGPTL4 concentrations in EPIC, we employed Cox proportional hazards models with age as the time scale and considered “minimally adjusted” (ie, stratified on sex, center of origin, and age at recruitment), “lifestyle adjusted” (ie, further adjusted for body mass index, alcohol consumption, smoking, physical activity, and education level), and “dietary factor adjusted” (ie, further adjusted for total daily energy intake, total daily red meat intake, total daily processed meat intake, total daily fiber intake, and total daily calcium intake) models. Prentice weights and robust variance were used to account for the case-cohort design. We repeated analyses stratified by sub-site (colon cancer, rectal cancer) and tested for heterogeneity by sub-site. Lag analyses were performed by repeating analyses excluding participants within the first 2 and 5 years of follow-up.

Analyses of pre-diagnostic ANGPTL4 concentrations and colorectal cancer risk in the UK Biobank cohort study used Cox proportional hazards models with age as the time scale. “Minimally adjusted” (ie, adjusted for sex, age at recruitment), “lifestyle adjusted” (ie, further adjusted for body mass index, alcohol consumption, smoking, physical activity, and education levels), and “dietary factor adjusted” (ie, further adjusted for red meat intake, processed meat intake, total daily energy intake, total daily calcium intake, and total daily fiber intake) models were employed. Analyses were repeated stratified on colorectal cancer subsite (colon cancer, rectal cancer) and “2 year” and “5 year” lag analyses were performed. Further information on cancer case definition and covariate classification across both EPIC and UK Biobank analyses is presented in the Supplementary Methods.

### Impact of ANGPTL4 loss-of-function on colon differential gene expression and gene set enrichment

To provide potential mechanistic insight into the effect of ANGPTL4 on precancerous molecular changes within the colon, we performed a phenome-wide association study of ANGPTL4 loss-of-function on differential gene expression in 445 normal colon tissue samples. For these analyses, we employed p. E40K (rs116843064), a variant that has been shown to abolish ANGPTL4 function.^51^ This variant was also used as a genetic instrument for circulating ANGPTL4 concentrations in drug-target MR analysis.

We then performed gene set enrichment analysis on genes whose expression was associated with p. E40K *(P *< .05) to identify biological pathways enriched among these genes using the Human MSigDB Collections Hallmark gene set.^52^ Gene set enrichment was performed using the fgsea R package with 1000 permutations.^53^ Further details on RNA-Seq and genotype processing, transcriptome-wide gene expression, and loss-of-function analyses are presented in the Supplementary Methods.

### ANGPTL4 tumor expression and all-cause mortality in colon cancer patients

To explore if ANGPTL4 is involved in cancer prognosis, we evaluated the association of *ANGPTL4* tumor expression with all-cause mortality in 481 colon cancer patients in TCGA. Read counts were normalized using the trimmed mean of M-values (TMM) method and then transformed to log2-counts per million reads. Cox proportional hazards models were employed with adjustment for age at diagnosis, gender, race, and American Joint Committee on Cancer (AJCC) pathologic stage. The time-to-event period was defined as the number of days between the initial diagnosis date and death or last follow-up. After excluding 16 participants with missing covariate data, there were 465 colon cancer patients. We also examined the association of *ANGPTL4* tumor expression with colorectal cancer-specific mortality using disease-specific survival (DSS) data defined and curated by the TCGA Clinical Data Resource.^54^ We did not explore the association of ANGPTL4 expression with rectal cancer because of the limited number of events (*N* = 23) in this analysis.

This study is reported as per the STROBE-MR reporting guidelines.^55^ All statistical analyses were performed using R version 4.3.1.

## Results

### Genetic instrument strength and validation analyses

Across the 5 drug targets, F-statistics for their instruments ranged from 306 to 3388, suggesting that genetic instruments were unlikely to suffer from weak instrument bias. Characteristics of genetic variants used to proxy drug targets are presented in Table 1. Estimates of r^2^ and F-statistics for each target are presented in Table S1.

**Table 1.** Characteristics of genetic variants used to proxy lipid-perturbing drug targets.

Target	SNP	Effect allele/non-effect allele	Effect allele frequency	Beta (SE)	*P*
*ANGPTL3*					
	rs10889352	C/T	0.35	−0.27 (0.01)	< 5 × 10^ − 324^
*ANGPTL4*					
	rs116843064	A/G	0.02	−0.35 (0.02)	5.70 × 10^ − 54^
*APOC3*					
	rs964184	C/G	0.88	−0.21 (0.01)	2.69 × 10^ − 65^
	rs187929675	T/C	0.01	−0.44 (0.04)	2.78 × 10^ − 29^
	rs141469619	A/G	0.99	−0.26 (0.04)	4.98 × 10^−13^
*CETP*					
	rs183130	T/C	0.33	−0.32 (0.01)	5.83 × 10^−136^
	rs158482	G/T	0.98	−0.31 (0.05)	1.81 × 10^−9^
*PCSK9*					
	rs11591147	T/G	0.02	−1.12 (0.02)	< 5 × 10^−324^
	rs472495	G/T	0.36	−0.15 (0.01)	5.72 × 10^−131^

Beta (SE) represents the change in circulating concentrations of the respective drug target per additional copy of the effect allele. Abbreviations: ANGPTL3 = Angiopoietin-like 3; ANGPTL4 = Angiopoietin-like 4; APOC3 = Apolipoprotein C3; CETP = Cholesteryl ester transfer protein; PCSK9 = Proprotein convertase subtilisin/kexin type 9; SNP = single-nucleotide polymorphism. Estimates were obtained from the UK Biobank for ANGPTL3, ANGPTL4, and PCSK9.

Findings from genetic instrument validation analyses using Mendelian randomization and colocalization were consistent with previously reported effects of approved and emerging medications on circulating lipid biomarkers reported in clinical trials (Table S2).

### Genetically proxied drug target perturbation and cancer risk

In analysis of 78 473 cases and 107 143 controls, there was evidence that genetically proxied circulating ANGPTL4 inhibition was associated with a reduced risk of colorectal cancer (odds ratio [OR] per SD decrease = 0.76, 95% confidence interval [CI] = 0.66 to 0.89, *P *= 5.52 × 10^−4^) (Table S3). There was a high posterior probability that circulating ANGPTL4 concentrations and colorectal cancer risk shared a causal variant within the *ANGPTL4* locus (PP_colocalization_ = 0.83). In analyses stratified on colorectal cancer subsite, there was weak evidence for differences in risk of colon cancer (OR = 0.86, 95% CI = 0.69 to 1.08, *P *= .19) and rectal cancer (OR = 0.64, 95% CI = 0.47 to 0.86, *P *= 3.20 × 10^−3^) (*P*_het_ = 0.12).

ANGPTL4 is a key regulator of plasma triglyceride levels, and therefore, we examined whether the association of genetically proxied ANGPTL4 inhibition was driven by reductions in circulating triglycerides. In MR analysis, we found little evidence of association of genetically proxied triglyceride concentrations with colorectal cancer risk in a primary IVW model (OR per unit decrease in log-transformed triglycerides = 1.04, 95% CI = 0.98 to 1.10; *P *= .22) or in pleiotropy-robust models (Table S4).

Genetically proxied ANGPTL4 inhibition was not associated with risk of 5 other cancers examined (FDR *P *< .05). Likewise, there was no evidence of association of genetically proxied ANGPTL3, APOC3, CETP, or PCSK9 inhibition with cancer risk (FDR *P *< .05) (Tables S5-S8). As such, subsequent analyses were restricted to ANGPTL4 and colorectal cancer and its subsites only.

### Association of pre-diagnostic ANGPTL4 concentrations and colorectal cancer risk

**Figure 2.**
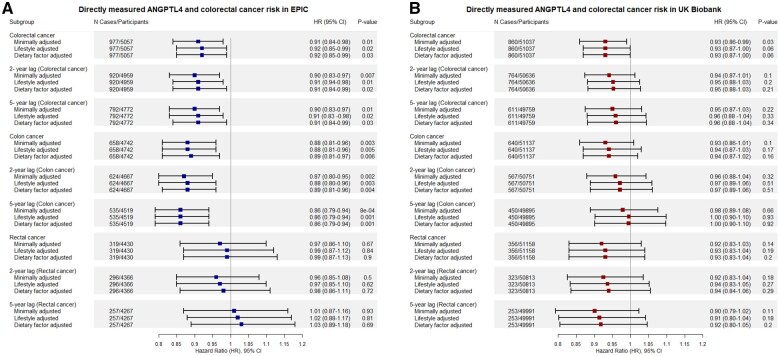
**A**) Minimally adjusted model was stratified on sex, center of origin, and age at recruitment. The lifestyle adjusted model was further adjusted for body mass index (BMI), alcohol (grams/day), smoking status (current, former, never smoker, unknown), physical activity index (inactive, moderately inactive, moderately active, active, missing), and highest level of education (not specified, none, primary, secondary, technical/professional, longer education). The dietary factor adjusted model was further adjusted for total daily energy intake, total daily red meat intake, total daily processed meat intake, total daily fiber intake, and total daily calcium intake. **B**) Minimally adjusted model was adjusted for sex and age at recruitment. The lifestyle adjusted model was further adjusted for BMI, alcohol consumption, smoking, physical activity, and education levels. The dietary factor adjusted model was further adjusted for red meat intake, processed meat intake, total daily energy intake, total daily calcium intake, and total daily fiber intake models were employed. 2-year lag = removed participants within the first 2 years of follow-up, 5-year lag = removed participants within the first 5 years of follow-up.

Case-cohort analyses in EPIC included 977 incident colorectal cancer cases and 4080 subcohort members (median 15.5 year follow-up). Compared with those in the lowest quartile, participants in the highest quartile of baseline circulating ANGPTL4 concentrations had higher levels of alcohol intake and were more likely to be a current smoker and to be physically active (Table 2). In the minimally adjusted multivariable regression model, we found evidence of a protective association of lower circulating ANGPTL4 concentrations (HR per log10 decrease = 0.91, 95% CI = 0.84 to 0.98, *P *= .01), consistent with genetic analyses. Findings did not materially change when further adjusted for lifestyle factors (HR per log10 decrease = 0.92, 95% CI = 0.85 to 0.99, *P *= .02) and dietary factors (HR = 0.92, 95% CI = 0.85 to 0.99, *P *= .03).The association of circulating ANGPTL4 concentrations with cancer risk did not differ by colorectal cancer subsite (HR colon cancer = 0.88, 95% CI = 0.81 to 0.96; HR rectal cancer = 0.97, 95% CI = 0.86 to 1.10; *P*_het_ = 0.20) and were consistent in lag analyses excluding participants within the first 2 and 5 years of follow-up (Figure 2A).

**Table 2.** Characteristics of EPIC case-cohort study participants by quartiles of circulating ANGPTL4 concentrations (N = 5057).

Characteristic	ANGPTL4 concentrations			
	Q1 (*N* = 1265)	Q2 (*N* = 1264)	Q3 (*N* = 1264)	Q4 (*N *= 1264)
Age at recruitment, y	52.1 (9.1)	52.8 (8.6)	52.7 (8.7)	52.4 (8.7)
Female (%)	875 (69.2)	790 (62.5)	742 (58.7)	639 (50.6)
Body mass index, kg/m^2^	26.7 (3.9)	27 (4.3)	26.9 (4.3)	27.2 (4.8)
Alcohol, g/day	10.8 (16.3)	13.1 (20.1)	13.9 (20.4)	16.5 (23.1)
Smoking (%)				
Never	687 (54.3)	626 (49.5)	600 (47.4)	604 (47.8)
Former	312 (24.7)	321 (25.4)	341 (27.0)	319 (25.2)
Current	266 (21.0)	317 (25.1)	324 (25.6)	341 (27.0)
Physical activity (%)				
Inactive	406 (32.1)	370 (29.2)	368 (29.1)	329 (26.0)
Moderately inactive	402 (31.8)	425 (33.6)	397 (31.4)	433 (34.3)
Moderately active	220 (17.4)	237 (18.8)	250 (19.8)	257 (20.3)
Active	219 (17.3)	217 (17.2)	233 (18.4)	243 (19.2)
Missing	18 (1.4)	15 (1.2)	16 (1.3)	3 (0.2)
Education level (%)				
None	236 (18.6)	199 (15.8)	191 (15.1)	174 (13.8)
Primary	480 (37.9)	492 (38.9)	452 (35.7)	456 (36.1)
Secondary	196 (15.5)	181 (14.3)	192 (15.2)	172 (13.6)
Technical/professional	169 (13.4)	212 (16.8)	197 (15.6)	219 (17.3)
Longer education	173 (13.7)	161 (12.7)	192 (15.2)	187 (14.8)
Not specified	11 (0.9)	19 (1.5)	40 (3.2)	55 (4.4)

Values are means and standard deviations for continuous variables and frequencies and percentages for categorical variables.

Prospective analyses in the UK Biobank included 860 incident colorectal cancer cases and 50 177 controls (median 14.2 year follow-up) (Table 3). Compared with those in the lowest quartile, participants in the highest quartile of baseline circulating ANGPTL4 concentrations had a higher mean BMI, lower levels of educational attainment, and spent less time in vigorous physical activity. In a model minimally adjusted for age and sex, we found evidence of a protective association of lower circulating ANGPTL4 concentrations with colorectal cancer risk (HR = 0.93, 95% CI = 0.86 to 0.99, *P *= .03). Findings did not materially change upon further adjustment for lifestyle (HR = 0.93, 95% CI = 0.87 to 1.00, *P *= .06) and dietary factors (HR = 0.93, 95% CI = 0.87 to 1.00, *P *= .06). Findings were similar across colon cancer (HR = 0.93, 95% CI = 0.86 to 1.01) and rectal cancer risk (HR = 0.92, 95% CI = 0.83 to 1.03; *P*_het_ = 0.87) and in “2 year” and “5 year” lag analyses (Figure 2B).

**Table 3.** Characteristics of UK Biobank prospective cohort study participants by quartiles of circulating ANGPTL4 concentrations (*N* = 51 291).

Characteristic	ANGPTL4 concentrations			
	Q1 (*N* = 12 825)	Q2 (*N* = 12 823)	Q3 (*N* = 12 821)	Q4 (*N* = 12 822)
Age at recruitment, y	54.7 (8.2)	56.4 (8.2)	57.4 (8.0)	58.6 (8.0)
Female (%)	7423 (57.9)	6886 (53.7)	6832 (53.3)	6532 (50.9)
Body mass index, kg/m^2^	26.1 (4.0)	27.1 (4.3)	27.8 (4.7)	28.9 (5.6)
Alcohol (%)				
Never	1029 (8.0)	997 (7.8)	1094 (8.5)	1305 (10.2)
Special occasions only	1353 (10.5)	1356 (10.6)	1497 (11.7)	1805 (14.1)
1 to 3 times a month	1336 (10.4)	1324 (10.3)	1410 (11.0)	1501 (11.7)
Once or twice a week	3175 (24.8)	3395 (26.5)	3451 (26.9)	3277 (25.6)
3 or 4 times a week	3143 (24.5)	3016 (23.5)	2865 (22.3)	2526 (19.7)
Daily or almost daily	2762 (21.5)	2704 (21.1)	2472 (19.3)	2380 (18.6)
Missing or unknown	27 (0.2)	31 (0.2)	32 (0.2)	28 (0.2)
Smoking (%)				
Never	7328 (57.1)	7097 (55.3)	6835 (53.3)	6500 (50.7)
Previous	4142 (32.3)	4412 (34.4)	4513 (35.2)	4801 (37.4)
Current	1307 (10.2)	1249 (9.7)	1412 (11.0)	1446 (11.3)
Unknown or missing	48 (0.4)	65 (0.5)	61 (0.4)	75 (0.6)
Physical activity (min)				
Walking	1039.0 (1094.2)	1039.6 (1080.2)	1040.7 (1085.7)	1012.9 (1078.2)
Moderate	925.0 (1211.0)	909.7 (1199.1)	952.2 (1244.2)	920.8 (1224.3)
Vigorous	712.3 (1222.0)	648.5 (1130.2)	661.4 (1227.2)	627.9 (1182.8)
Education level (%)				
None	1638 (12.8)	1957 (15.3)	2349 (18.3)	3106 (24.2)
CSEs or equivalent	705 (5.5)	673 (5.2)	704 (5.5)	666 (5.2)
O levels/GCSEs or equivalent	2556 (19.9)	2645 (20.6)	2798 (21.8)	2655 (20.7)
NVQ or HND or HNC or equivalent	780 (6.1)	809 (6.3)	856 (6.7)	952 (7.4)
A levels/AS levels or equivalent	1515 (11.8)	1503 (11.7)	1361 (10.6)	1261 (9.8)
College or University degree	4851 (37.8)	4409 (34.4)	3918 (30.6)	3250 (25.3)
Other professional qualifications	630 (4.9)	671 (5.2)	683 (5.3)	717 (5.6)
Unknown or missing	150 (1.2)	156 (1.2)	152 (1.2)	215 (1.7)

Values are means and standard deviations for continuous variables and frequencies and percentages for categorical variables. Abbreviations: CSE = Certificate of Secondary Education, GCSE = General Certificates of Secondary Education, NVQ = National Vocational Qualification, HND = Higher National Diploma, HNC = Higher National Certificate, A levels = Advanced Level Qualification, AS = Advanced Subsidiary Level Qualification.

### Impact of ANGPTL4 loss-of-function on colon differential gene expression and gene set enrichment

**Figure 3.**
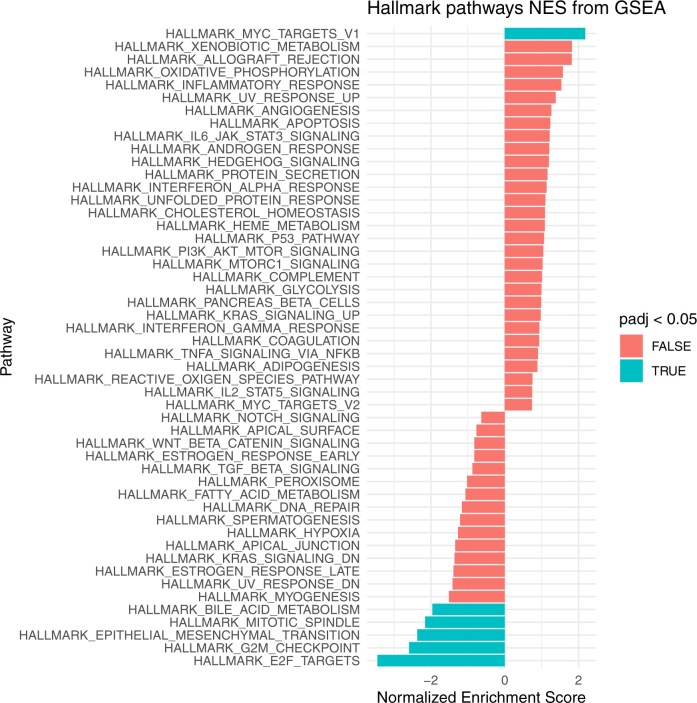
GSEA = Gene-set enrichment analysis.

In gene-level analysis, we did not find evidence for an association of the loss-of-function p. E40K variant with differential gene expression after correcting for multiple testing (FDR *P *< .05) (Table S9). However, when exploring pathway-level enrichment using gene set enrichment analysis, differentially expressed genes (*P *< .05) were strongly enriched for 6 Hallmark gene sets (FDR *P *< .05). Down-regulated gene sets included those implicated in cellular proliferation (ie, targets of the E2F family of transcription factors, genes involved in the cell cycle G2/M checkpoint, and genes involved in mitotic spindle assembly), epithelial-mesenchymal transition (EMT), and bile acid metabolism (Figure 3, Table S10). There was one up-regulated gene set implicated in cellular proliferation (ie, genes regulated by the oncogenic MYC pathway).

### Colon tumor ANGPTL4 expression and all-cause mortality

After a median follow-up of 1.8 (IQR 1.0-3.0) years of 465 colon cancer patients, 98 deaths were recorded, including 41 colorectal cancer deaths. In multivariable-adjusted Cox proportional hazards models, lower colon tumor *ANGPTL4* gene expression was associated with reduced risk of all-cause mortality (HR per log2 decrease = 0.85, 95% CI = 0.73 to 0.99; *P *= .04). We also found that lower colon tumor *ANGPTL4* expression was associated with reduced risk of colorectal cancer-specific mortality (HR = 0.66, 95% CI = 0.50 to 0.87; *P *= 2.92 × 10^−3^).

## Discussion

Through triangulation of evidence across proteogenomic, observational, and molecular epidemiological analyses, we prioritize ANGPTL4 as a potential therapeutic target for colorectal cancer prevention. In combined drug-target Mendelian randomization and colocalization analyses of 78 473 cases and 107 143 controls, genetically proxied ANGPTL4 inhibition was associated with a reduced risk of colorectal cancer. In replication analyses using independent prospective analysis of 977 incident colorectal cancer cases and 4080 non-cases in EPIC and 860 incident colorectal cancer cases and 50 177 controls in the UK Biobank, directly measured lower circulating ANGPTL4 concentrations were associated with reduced colorectal cancer risk. In gene set enrichment analysis of differential gene expression in 445 normal colon tissue samples, ANGPTL4 loss-of-function was associated with down-regulation of several cancer-related gene-sets, providing mechanistic insight into anti-tumorigenic properties of ANGPTL4. Finally, in analysis of 465 colon cancer patients, lower *ANGPTL4* expression in colon tumor tissue was associated with a reduced risk of all-cause mortality. Collectively, these findings provide strong and consistent support for a potential role of ANGPTL4 in colorectal tumorigenesis. We found limited evidence to support differences in the association of ANGPTL4 with colon and rectal cancer risk in genetic studies though these analyses were likely underpowered to detect heterogeneity. In contrast, in analyses examining the association of directly measured ANGPTL4 across both EPIC and UK Biobank we found no evidence of heterogeneity of association, suggesting that limited evidence of heterogeneity in genetic studies could reflect a chance finding.

ANGPTL4 is a ubiquitously expressed glycoprotein that inhibits lipoprotein lipase and modulates fatty acid uptake in adipose and oxidative tissue.^56-60^ As a key regulator of triglyceride clearance, ANGPTL4 has emerged as an attractive therapeutic target for reducing triglyceride levels and adverse cardiovascular events.^61^^,^^62^ This is supported by human genetic evidence that loss-of-function variants in *ANGPTL4* are associated with lower plasma triglycerides and reduced coronary artery disease risk.^63^ In addition, human genetic inactivation of *ANGPTL4* has been shown to improve glucose homeostasis and reduce type 2 diabetes risk.^64^ At least 2 pharmacological ANGPTL4 inhibitors are currently under Phase II clinical trial evaluation for their efficacy in lowering plasma triglycerides and reducing cardiovascular events.^65^^,^^66^

Our findings potentially implicating ANGPTL4 in colorectal cancer development recapitulate insights from preclinical studies. For example, *ANGPTL4* knockdown has been shown to inhibit proliferation, promote apoptosis, and suppress migration in colorectal cancer cell lines and to reduce colorectal tumor size in xenograft mouse models.^67^^,^^68^ Recombinant ANGPTL4 has been reported to promote colon cancer growth by impairing CD8+ T cell activity in mice.^69^ Recently, ANGPTL4 suppression has been shown to reprogram endothelial cell metabolism and inhibit angiogenesis, providing another mechanism through which ANGPTL4 may influence carcinogenesis.^70^ Interestingly, prostaglandin E2, a putative key mediator of the effect of COX-2 on colorectal cancer, has also been reported to promote colorectal carcinoma cell proliferation via ANGPTL4 under hypoxic conditions.^71^^,^^72^

Consistent with prior reports, we did not find evidence of an association of genetically proxied triglyceride concentrations with colorectal cancer risk, suggesting that the association between ANGPTL4 and colorectal cancer risk is mediated via pathways independent of triglyceride lowering.^26^^,^^73^ In gene set enrichment analysis, ANGPTL4 loss-of-function lead to down-regulation of several biological pathways implicated in colon carcinogenesis including cellular proliferation, bile acid metabolism, and the EMT. For example, bile acids have been shown to promote colon cancer by damaging colonic epithelial cells, and inducing reactive oxygen species production, genomic destabilization, and apoptosis resistance.^74^ In addition, the EMT has been reported to play an important role in colorectal cancer progression, metastasis, and drug resistance, and preclinical studies have suggested the efficacy of pharmacological perturbation of markers of the EMT in colorectal cancer.^75^ Our findings thus validate and extend insights from preclinical models and can help to guide future work investigating mechanisms underpinning the effect of ANGPTL4 on colorectal cancer development.

Contrary to some prior studies, we found little evidence to support associations of other lipid-perturbing targets (eg, PCSK9, CETP) with cancer risk (eg, breast, prostate, head and neck).^27^^,^^28^^,^^76^ The absence of or inconsistent application of colocalization analysis in some prior studies complicates assessment of whether discordance between findings reflects the presence of confounding by LD in previous studies, differences in instrument construction strategy across studies, or chance. Nonetheless, our findings suggesting little evidence of association of genetically proxied ANGPTL3, APOC3, CETP, and PCSK9 inhibition with cancer risk may help to deprioritize further evaluation of these proteins as intervention targets for cancer prevention.

Strengths of this study include use of a triangulation framework leveraging genetic and conventional epidemiological approaches to strengthen causal inference. Notably, the consistency of findings across drug-target Mendelian randomization and conventional epidemiological analysis using independent cohort studies, both of which may be susceptible to unrelated sources of bias, permitted us to increase confidence in our conclusions relating circulating ANGPTL4 to colorectal cancer risk.^77^ By leveraging gene expression data from normal and cancerous colon tissue samples we gained potential mechanistic insight into the effect of ANGPTL4 on early precancerous changes in the colon and extended exploration of the role of ANGPTL4 to mortality among colon cancer patients, supporting a possible role of this target across the carcinogenesis spectrum.

There are several limitations to this analysis. First, the validity of findings from *cis*-MR and gene expression analyses is dependent upon both exchangeability (ie, no confounding of the instrument-outcome association) and exclusion restriction (ie, no direct effect of the instrument on the outcome). Although various sensitivity analyses were performed to evaluate the robustness of findings to violations of both assumptions, these are unverifiable. Nonetheless, the use of the E40K predicted loss-of-function variant, that has been shown to directly influence ANGPTL4 protein function, to proxy ANGPTL4 inhibition along with evidence that circulating ANGPTL4 and colorectal cancer risk share a causal variant within the *ANGPTL4* locus should minimize the likelihood that these assumptions have been violated.^51^ Second, conventional observational analyses performed in EPIC, the UK Biobank, and TCGA assume the absence of confounding, measurement error, and reverse causation though lag analyses in EPIC were consistent with the primary analysis. Third, genetic and conventional observational analyses are restricted to examining on-target (ie, target-mediated) effects of medications. Fourth, statistical power was likely limited in drug-target MR analyses of less common cancer subtypes. Fifth, genetic and conventional observational analyses were primarily performed in participants of European ancestry and, therefore, the generalizability of these findings to non-European populations is unclear. Sixth, we were unable to explore the association of both *ANGPTL4* loss-of-function with differential gene expression in normal rectal tissue because of the absence of suitable data in this tissue and *ANGPTL4* expression in rectal tumor samples with all-cause mortality due to the limited number of events in this dataset.

Colorectal cancer is the third most common cancer globally, accounting for more than 900 000 deaths in 2022.^78^^,^^79^ Aspirin and nonsteroidal anti-inflammatory drugs can be used to lower colorectal cancer risk in high-risk populations (eg, individuals with Lynch syndrome, familial adenomatous polyposis) but the increased risk of gastrointestinal bleeding on these medications limit their wider use.^80^ There is therefore a need for identification of novel safe and effective chemoprevention agents for colorectal cancer to reduce the burden from this disease. Our findings leveraging genetic, observational, and molecular epidemiological designs recapitulate insights from preclinical studies indicating a protective effect of ANGPTL4 inhibition in colorectal cancer risk. Further work validating findings in human studies and clarifying potential mechanisms of effect will guide further assessment of the viability of ANGPTL4 inhibition as a therapeutic strategy for cancer prevention. In addition, investigation of the role of ANGPTL4 in colorectal carcinogenesis in non-European populations will permit evaluation of the generalizability of these findings to other ancestries. Finally, ongoing clinical trials investigating pharmacological ANGPTL4 inhibition for CVD present another opportunity to explore potential cancer preventive properties of these medications.

## Conclusion

In conclusion, our comprehensive proteogenomic and observational analyses suggest a protective role of lowering circulating ANGPTL4 concentrations in colorectal cancer risk. These findings provide human validation to insights from preclinical studies and support the further evaluation of ANGPTL4 as a potential therapeutic target for colorectal cancer prevention.

## Supplementary Material

djaf137_Supplementary_Data

## Data Availability

Data supporting the findings of this study are available within the paper and its supplementary information files. Summary genetic association data on breast, ovarian, and prostate cancer can be obtained from the GWAS Catalog (accession numbers: GCST004988, 28346442, 29892016). Summary genetic association data on head and neck cancer will be posted on the GWAS Catalog with publication of Ebrahimi et al. medRxiv, 2024 (https://www.medrxiv.org/content/10.1101/2024.11.18.24317473v1). Summary genetic association data on colorectal cancer were obtained by submitting a data usage proposal to The Genetics and Epidemiology of Colorectal Cancer Consortium (GECCO) (https://research.fredhutch.org/peters/en/genetics-and-epidemiology-of-colorectal-cancer-consortium.html). Data from the EPIC study can be obtained by submitting a scientific proposal form (PF1) to the data access committee (https://epic.iarc.fr/).

## References

[1] Fahy E , Cotter D, Sud M, Subramaniam S. Lipid classification, structures and tools. Biochim Biophys Acta. 2011;1811:637-647. doi:10.1016/j.bbalip.2011.06.009 21704189 PMC3995129

[2] Ackerman D , Simon MC. Hypoxia, lipids, and cancer: surviving the harsh tumor microenvironment. Trends Cell Biol. 2014;24:472-478. doi:10.1016/j.tcb.2014.06.001 24985940 PMC4112153

[3] Broadfield LA , Pane AA, Talebi A, Swinnen JV, Fendt SM. Lipid metabolism in cancer: new perspectives and emerging mechanisms. Dev Cell. 2021;56:1363-1393. doi:10.1016/j.devcel.2021.04.013 33945792

[4] Liu R , Huang Y. Lipid signaling in tumorigenesis. Mol Cell Pharmacol. 2014;6:1-9. 25741396 PMC4346139

[5] Capece D , Franzoso G. Rewired lipid metabolism as an actionable vulnerability of aggressive colorectal carcinoma. Mol Cell Oncol. 2022;9:2024051. 10.1080/23723556.2021.2024051 35252551 PMC8890390

[6] Snaebjornsson MT , Janaki-Raman S, Schulze A. Greasing the wheels of the cancer machine: the role of lipid metabolism in cancer. Cell Metab. 2020;31:62-76. 10.1016/j.cmet.2019.11.010 31813823

[7] Koundouros N , Poulogiannis G. Reprogramming of fatty acid metabolism in cancer. Br J Cancer. 2020;122:4-22. 10.1038/s41416-019-0650-z 31819192 PMC6964678

[8] Corn KC , Windham MA, Rafat M. Lipids in the tumor microenvironment: from cancer progression to treatment. Prog Lipid Res. 2020;80:101055. 10.1016/j.plipres.2020.101055 32791170 PMC7674189

[9] Juarez D , Fruman DA. Targeting the mevalonate pathway in cancer. Trends Cancer. 2021;7:525-540. 10.1016/j.trecan.2020.11.008 33358111 PMC8137523

[10] Tan MJ , Teo Z, Sng MK, Zhu P, Tan NS. Emerging roles of angiopoietin-like 4 in human cancer. Mol Cancer Res. 2012;10:677-688. 10.1158/1541-7786.Mcr-11-0519 22661548

[11] Wang H , Guo Q, Wang M, Liu C, Tian Z. PCSK9 promotes tumor cell proliferation and migration by facilitating CCL25 secretion in esophageal squamous cell carcinoma. Oncol Lett. 2023;26:500. 10.3892/ol.2023.14086 37854863 PMC10579978

[12] Poynter JN , Gruber SB, Higgins PD, et al Statins and the risk of colorectal cancer. N Engl J Med. 2005;352:2184-2192. 10.1056/NEJMoa043792 15917383

[13] Oza PP , Kashfi K. The evolving landscape of PCSK9 inhibition in cancer. Eur J Pharmacol. 2023;949:175721. 10.1016/j.ejphar.2023.175721 37059376 PMC10229316

[14] Carbone C , Piro G, Merz V, et al Angiopoietin-like proteins in angiogenesis, inflammation and cancer. Int J Mol Sci. 2018;19:431. 10.3390/ijms19020431 29389861 PMC5855653

[15] Koyama T , Ogawara K, Kasamatsu A, et al ANGPTL3 is a novel biomarker as it activates ERK/MAPK pathway in oral cancer. Cancer Med. 2015;4:759-769. 10.1002/cam4.418 25644496 PMC4430268

[16] Zhong L , Tang L, He X. Angiopoietin-like 3 (ANGPTL3) drives cell proliferation, migration and angiogenesis in cervical cancer via binding to integrin alpha v beta 3. Bioengineered. 2022;13:2971-2980. 10.1080/21655979.2021.2024951 35038961 PMC8974177

[17] Archibugi L , Arcidiacono PG, Capurso G. Statin use is associated to a reduced risk of pancreatic cancer: a meta-analysis. Dig Liver Dis. 2019;51:28-37. 10.1016/j.dld.2018.09.007 30314951

[18] Irvin S , Clarke MA, Trabert B, Wentzensen N. Systematic review and meta-analysis of studies assessing the relationship between statin use and risk of ovarian cancer. Cancer Causes Control. 2020;31:869-879. 10.1007/s10552-020-01327-8 32685996 PMC7484024

[19] Mondul AM , Joshu CE, Barber JR, et al Longer-term lipid-lowering drug use and risk of incident and fatal prostate cancer in Black and White men in the ARIC Study. Cancer Prev Res (Phila). 2018;11:779-788. 10.1158/1940-6207.Capr-17-0396 30327368 PMC6289799

[20] Ren QW , Yu SY, Teng TK, et al Statin associated lower cancer risk and related mortality in patients with heart failure. Eur Heart J. 2021;42:3049-3059. 10.1093/eurheartj/ehab325 34157723 PMC8380061

[21] Tuyet Kristensen D , Kisbye Øvlisen A, Hjort Kyneb Jakobsen L, et al Use of statins and risk of myeloproliferative neoplasms: a Danish nationwide case-control study. Blood Adv. 2023;7:3450-3457. 10.1182/bloodadvances.2023009784 36877642 PMC10362262

[22] Lawlor DA , Davey Smith G, Kundu D, Bruckdorfer KR, Ebrahim S. Those confounded vitamins: what can we learn from the differences between observational versus randomised trial evidence? Lancet. 2004;363:1724-1727. 10.1016/s0140-6736(04)16260-0 15158637

[23] Phillips AN , Smith GD. How independent are “independent” effects? Relative risk estimation when correlated exposures are measured imprecisely. J Clin Epidemiol. 1991;44:1223-1231. 10.1016/0895-4356(91)90155-3 1941017

[24] Sattar N , Preiss D. Reverse causality in cardiovascular epidemiological research: more common than imagined? Circulation. 2017;135:2369-2372. 10.1161/circulationaha.117.028307 28606949

[25] Lipigon reports a statistically confirmed reduction of target protein ANGPTL4 after repeated treatment with Lipisense^®^. 2024. Accessed April 15, 2025. https://www.lipigon.se/en/investors/press-releases/? slug=lipigon-reports-a-statistically-confirmed-reduction-of-targe-53815

[26] Cornish AJ , Law PJ, Timofeeva M, et al Modifiable pathways for colorectal cancer: a mendelian randomisation analysis. Lancet Gastroenterol Hepatol. 2020;5:55-62. 10.1016/s2468-1253(19)30294-8 31668584 PMC7026696

[27] Fang S , Yarmolinsky J, Gill D, et al; PRACTICAL Consortium. Association between genetically proxied PCSK9 inhibition and prostate cancer risk: a Mendelian randomisation study. PLoS Med. 2023;20:e1003988. 10.1371/journal.pmed.1003988 36595504 PMC9810198

[28] Gormley M , Yarmolinsky J, Dudding T, et al Using genetic variants to evaluate the causal effect of cholesterol lowering on head and neck cancer risk: a Mendelian randomization study. PLoS Genet. 2021;17:e1009525. 10.1371/journal.pgen.1009525 33886544 PMC8096036

[29] Ioannidou A , Watts EL, Perez-Cornago A, et al; PRACTICAL consortium, CRUK, BPC3, CAPS, PEGASUS. The relationship between lipoprotein A and other lipids with prostate cancer risk: a multivariable Mendelian randomisation study. PLoS Med. 2022;19:e1003859. 10.1371/journal.pmed.1003859 35085228 PMC8794090

[30] Johnson KE , Siewert KM, Klarin D, et al; VA Million Veteran Program. The relationship between circulating lipids and breast cancer risk: a Mendelian randomization study. PLoS Med. 2020;17:e1003302. 10.1371/journal.pmed.1003302 32915777 PMC7485834

[31] Yarmolinsky J , Bull CJ, Vincent EE, et al Association between genetically proxied inhibition of HMG-CoA reductase and epithelial ovarian cancer. JAMA. 2020;323:646-655. 10.1001/jama.2020.0150 32068819 PMC7042851

[32] Ebrahimi E , Sangphukieo A, Park H, et al; HEADSpAcE Consortium. Cross-ancestral GWAS identifies 29 novel variants across head and neck cancer subsites. medRxiv 2024.11.18.24317473. 10.1101/2024.11.18.24317473, 2024, preprint: not peer reviewed. PMC12491539 41038832

[33] Fernandez-Rozadilla C , Timofeeva M, Chen ZJ, et al Deciphering colorectal cancer genetics through multi-omic analysis of 100,204 cases and 154,587 controls of European and east Asian ancestries. Nat Genet. 2023;55:89-99. 10.1038/s41588-022-01222-9 36539618 PMC10094749

[34] Michailidou K , Lindström S, Dennis J, et al; ConFab/AOCS Investigators. Association analysis identifies 65 new breast cancer risk loci. Nature. 2017;551:92-94. 10.1038/nature24284 29059683 PMC5798588

[35] Phelan CM , Kuchenbaecker KB, Tyrer JP, et al OPAL study group. Identification of 12 new susceptibility loci for different histotypes of epithelial ovarian cancer. Nat Genet. 2017;49:680-691. 10.1038/ng.3826 28346442 PMC5612337

[36] Schumacher FR , Al Olama AA, Berndt SI, et al; Genetic Associations and Mechanisms in Oncology (GAME-ON)/Elucidating Loci Involved in Prostate Cancer Susceptibility (ELLIPSE) Consortium. Association analyses of more than 140,000 men identify 63 new prostate cancer susceptibility loci. Nat Genet. 2018;50:928-936. 10.1038/s41588-018-0142-8 29892016 PMC6568012

[*37] Graham SE , Clarke SL, Wu KH, et al; Global Lipids Genetics Consortium. The power of genetic diversity in genome-wide association studies of lipids. Nature. 2021;600:675-679. 10.1038/s41586-021-04064-3 34887591 PMC8730582

[38] Riboli E , Kaaks R. The EPCI Project: European Prospective Investigation into Cancer and Nutrition. Int J Epidemiol 1997; 26:S6-S14. 9126529 10.1093/ije/26.suppl_1.s6

[39] Díez-Obrero V , Dampier CH, Moratalla-Navarro F, et al Genetic effects on transcriptome profiles in colon epithelium provide functional insights for genetic risk loci. Cell Mol Gastroenterol Hepatol. 2021;12:181-197. 10.1016/j.jcmgh.2021.02.003 33601062 PMC8102177

[40] Weinstein JN , Collisson EA, Mills GB, et al; Cancer Genome Atlas Research Network. The Cancer Genome Atlas Pan-Cancer analysis project. Nat Genet. 2013;45:1113-1120. 10.1038/ng.2764 24071849 PMC3919969

[41] Ferkingstad E , Sulem P, Atlason BA, et al Large-scale integration of the plasma proteome with genetics and disease. Nat Genet. 2021;53:1712-1721. 10.1038/s41588-021-00978-w 34857953

[42] Sun BB , Chiou J, Traylor M, et al; Regeneron Genetics Center. Plasma proteomic associations with genetics and health in the UK Biobank. Nature. 2023;622:329-338. 10.1038/s41586-023-06592-6 37794186 PMC10567551

[43] Bowden J , Del Greco MF, Minelli C, Davey Smith G, Sheehan N, Thompson J. A framework for the investigation of pleiotropy in two-sample summary data Mendelian randomization. Stat Med. 2017;36:1783-1802. 10.1002/sim.7221 28114746 PMC5434863

[44] Raal FJ , Rosenson RS, Reeskamp LF, et al; ELIPSE HoFH Investigators. Evinacumab for homozygous familial hypercholesterolemia. N Engl J Med. 2020;383:711-720. 10.1056/NEJMoa2004215 32813947

[45] Sabatine MS , Giugliano RP, Keech AC, et al; FOURIER Steering Committee and Investigators. Evolocumab and clinical outcomes in patients with cardiovascular disease. N Engl J Med. 2017;376:1713-1722. 10.1056/NEJMoa1615664 28304224

[46] Gaudet D , Clifton P, Sullivan D, et al RNA Interference therapy targeting apolipoprotein C-III in hypertriglyceridemia. NEJM Evid. 2023;2:EVIDoa2200325. 10.1056/EVIDoa2200325 38320498

[47] Bowman L , Hopewell JC, Chen F, et al; HPS3/TIMI55–REVEAL Collaborative Group. Effects of anacetrapib in patients with atherosclerotic vascular disease. N Engl J Med. 2017;377:1217-1227. 10.1056/NEJMoa1706444 28847206

[48] Bowden J , Davey Smith G, Burgess S. Mendelian randomization with invalid instruments: effect estimation and bias detection through Egger regression. Int J Epidemiol. 2015;44:512-525. 10.1093/ije/dyv080 26050253 PMC4469799

[49] Bowden J , Davey Smith G, Haycock PC, Burgess S. Consistent estimation in Mendelian randomization with some invalid instruments using a weighted median estimator. Genet Epidemiol. 2016;40:304-314. 10.1002/gepi.21965 27061298 PMC4849733

[50] Hartwig FP , Davey Smith G, Bowden J. Robust inference in summary data Mendelian randomization via the zero modal pleiotropy assumption. Int J Epidemiol. 2017;46:1985-1998. 10.1093/ije/dyx102 29040600 PMC5837715

[51] Yin W , Romeo S, Chang S, Grishin NV, Hobbs HH, Cohen JC. Genetic variation in ANGPTL4 provides insights into protein processing and function. J Biol Chem. 2009;284:13213-13222. 10.1074/jbc.M900553200 19270337 PMC2676053

[52] Liberzon A , Birger C, Thorvaldsdóttir H, Ghandi M, Mesirov JP, Tamayo P. The Molecular Signatures Database (MSigDB) hallmark gene set collection. Cell Syst. 2015;1:417-425. 10.1016/j.cels.2015.12.004 26771021 PMC4707969

[53] Korotkevich G , Sukhov V, Budin N, Shpak B, Artyomov MN, Sergushichev A. Fast gene set enrichment analysis. bioRxiv 060012. 10.1101/060012, 2021, preprint: not peer reviewed.

[54] Liu J , Lichtenberg T, Hoadley KA, et al; Cancer Genome Atlas Research Network. An integrated TCGA pan-cancer clinical data resource to drive high-quality survival outcome analytics. Cell. 2018;173:400-416.e11. 10.1016/j.cell.2018.02.052 29625055 PMC6066282

[55] Skrivankova VW , Richmond RC, Woolf BAR, et al Strengthening the reporting of observational studies in epidemiology using Mendelian randomization: the STROBE-MR Statement. JAMA. 2021;326:1614-1621. 10.1001/jama.2021.18236 34698778

[56] Yang YH , Wang Y, Lam KS, et al Suppression of the Raf/MEK/ERK signaling cascade and inhibition of angiogenesis by the carboxyl terminus of angiopoietin-like protein 4. Arterioscler Thromb Vasc Biol. 2008;28:835-840. 10.1161/atvbaha.107.157776 18340008

[57] Ge H , Yang G, Yu X, Pourbahrami T, Li C. Oligomerization state-dependent hyperlipidemic effect of angiopoietin-like protein 4. J Lipid Res. 2004;45:2071-2079. 10.1194/jlr.M400138-JLR200 15292369

[58] Mandard S , Zandbergen F, van Straten E, et al The fasting-induced adipose factor/angiopoietin-like protein 4 is physically associated with lipoproteins and governs plasma lipid levels and adiposity. J Biol Chem. 2006;281:934-944. 10.1074/jbc.M506519200 16272564

[59] Sukonina V , Lookene A, Olivecrona T, Olivecrona G. Angiopoietin-like protein 4 converts lipoprotein lipase to inactive monomers and modulates lipase activity in adipose tissue. Proc Natl Acad Sci U S A. 2006;103:17450-17455. 10.1073/pnas.0604026103 17088546 PMC1859949

[60] Xu A , Lam MC, Chan KW, et al Angiopoietin-like protein 4 decreases blood glucose and improves glucose tolerance but induces hyperlipidemia and hepatic steatosis in mice. Proc Natl Acad Sci U S A. 2005;102:6086-6091. 10.1073/pnas.0408452102 15837923 PMC1087912

[61] Aryal B , Price NL, Suarez Y, Fernández-Hernando C. ANGPTL4 in metabolic and cardiovascular disease. Trends Mol Med. 2019;25:723-734. 10.1016/j.molmed.2019.05.010 31235370 PMC6779329

[62] Kersten S. Role and mechanism of the action of angiopoietin-like protein ANGPTL4 in plasma lipid metabolism. J Lipid Res. 2021;62:100150. 10.1016/j.jlr.2021.100150 34801488 PMC8666355

[63] Dewey FE , Gusarova V, O'Dushlaine C, et al Inactivating variants in ANGPTL4 and risk of coronary artery disease. N Engl J Med. 2016;374:1123-1133. 10.1056/NEJMoa1510926 26933753 PMC4900689

[64] Gusarova V , O'Dushlaine C, Teslovich TM, et al Genetic inactivation of ANGPTL4 improves glucose homeostasis and is associated with reduced risk of diabetes. Nat Commun. 2018;9:2252. 10.1038/s41467-018-04611-z 29899519 PMC5997992

[65] The Swedish Medical Products Agency approves Lipigon's phase II study with Lipisense^®^. Accessed April 15, 2025. https://www.lipigon.se/en/investors/press-releases/? slug=the-swedish-medical-products-agency-approves-lipigon-s-phase-96913

[66] Marea therapeutics launches with $190 million to accelerate a new generation of medicines for cardiometabolic diseases. 2024. Accessed October 18, 2024. https://www.businesswire.com/news/home/20240617273647/en/Marea-Therapeutics-Launches-with-190-Million-to-Accelerate-a-New-Generation-of-Medicines-for-Cardiometabolic-Diseases

[67] Mizuno S , Seishima R, Yamasaki J, et al Angiopoietin-like 4 promotes glucose metabolism by regulating glucose transporter expression in colorectal cancer. J Cancer Res Clin Oncol. 2022;148:1351-1361. 10.1007/s00432-022-03960-z 35195748 PMC11800850

[68] Wen L , Zhang Y, Yang B, Han F, Ebadi AG, Toughani M. Knockdown of angiopoietin-like protein 4 suppresses the development of colorectal cancer. Cell Mol Biol (Noisy-le-Grand). 2020;66:117-124. 33040824

[69] Ding S , Lin Z, Zhang X, et al Deficiency of angiopoietin-like 4 enhances CD8(+) T cell bioactivity via metabolic reprogramming for impairing tumour progression. Immunology. 2023;170:28-46. 10.1111/imm.13650 37094816

[70] Chaube B , Citrin KM, Sahraei M, et al Suppression of angiopoietin-like 4 reprograms endothelial cell metabolism and inhibits angiogenesis. Nat Commun. 2023;14:8251. 10.1038/s41467-023-43900-0 38086791 PMC10716292

[71] Kim SH , Park YY, Kim SW, Lee JS, Wang D, DuBois RN. ANGPTL4 induction by prostaglandin E2 under hypoxic conditions promotes colorectal cancer progression. Cancer Res. 2011;71:7010-7020. 10.1158/0008-5472.Can-11-1262 21937683 PMC3217078

[72] Wang D , Buchanan FG, Wang H, Dey SK, DuBois RN. Prostaglandin E2 enhances intestinal adenoma growth via activation of the Ras-mitogen-activated protein kinase cascade. Cancer Res. 2005;65:1822-1829. 10.1158/0008-5472.Can-04-3671 15753380

[73] Iwagami M , Goto A, Katagiri R, et al Blood lipids and the risk of colorectal cancer: Mendelian randomization analyses in the Japanese Consortium of genetic epidemiology studies. Cancer Prev Res (Phila). 2022;15:827-836. 10.1158/1940-6207.Capr-22-0146 36040498

[74] Nguyen TT , Ung TT, Kim NH, Jung YD. Role of bile acids in colon carcinogenesis. World J Clin Cases. 2018;6:577-588. 10.12998/wjcc.v6.i13.577 30430113 PMC6232560

[75] Zhang N , Ng AS, Cai S, Li Q, Yang L, Kerr D. Novel therapeutic strategies: targeting epithelial-mesenchymal transition in colorectal cancer. Lancet Oncol. 2021;22:e358-e368. 10.1016/s1470-2045(21)00343-0 34339656

[76] Nowak C , Ärnlöv J. A Mendelian randomization study of the effects of blood lipids on breast cancer risk. Nat Commun. 2018;9:3957. 10.1038/s41467-018-06467-9 30262900 PMC6160471

[77] Lawlor DA , Tilling K, Davey Smith G. Triangulation in aetiological epidemiology. Int J Epidemiol. 2016;45:1866-1886. 10.1093/ije/dyw314 28108528 PMC5841843

[78] Bray F , Laversanne M, Sung H, et al Global cancer statistics 2022: GLOBOCAN estimates of incidence and mortality worldwide for 36 cancers in 185 countries. CA Cancer J Clin. 2024;74:229-263. 10.3322/caac.21834 38572751

[79] Morgan E , Arnold M, Gini A, et al Global burden of colorectal cancer in 2020 and 2040: incidence and mortality estimates from GLOBOCAN. Gut. 2023;72:338-344. 10.1136/gutjnl-2022-327736 36604116

[80] Katona BW , Weiss JM. Chemoprevention of colorectal cancer. Gastroenterology. 2020;158:368-388. 10.1053/j.gastro.2019.06.047 31563626 PMC6981249

