# Supplementary material for: Proteogenomic and observational evidence implicate ANGPTL4 as a potential therapeutic target for colorectal cancer prevention
Source: J Natl Cancer Inst. 2025 Jun 13;117(9):1836–47. doi: 10.1093/jnci/djaf137 (PMC12415964; doi:10.1093/jnci/djaf137)
Supplement: djaf137_Supplementary_Data [file djaf137_supplementary_data.zip › ANGPTL4 CRC Supplementary Materials 18.11.24 -R (Clean).docx]

**Study populations**

Overall and estrogen receptor (ER)-stratified breast cancer risk in up to 122,977 cases and 105,974 controls were obtained from the Breast Cancer Association Consortium (BCAC)^1^. Summary genetic association estimates for overall and site-specific colorectal cancer risk in up to 78,473 cases and 107,143 controls were obtained from an analysis of the Genetics and Epidemiology of Colorectal Cancer Consortium (GECCO), ColoRectal Transdisciplinary Study (CORECT), and Colon Cancer Family Registry (CCFR)^2^. Summary genetic association estimates for prostate cancer risk in up to 79,148 cases and 61,106 controls were obtained from the Prostate Cancer Association Group to Investigate Cancer Associated Alterations in the Genome (PRACTICAL) consortium^3^. Summary genetic association data for overall and subtype-specific head and neck cancer in up to 13,554 cases and 32,914 controls were obtained from a prior GWAS^4^. Summary genetic association data on overall and histotype-specific epithelial ovarian cancer risk were obtained from 25,509 cases and 40,941 controls from the Ovarian Cancer Association Consortium (OCAC)^5^. Further information on statistical analysis, imputation, and quality control measures for these studies is available in the original publications.

**Drug-target Mendelian randomization**

Instrumental variable assumptions:

Drug-target Mendelian randomization can generate valid tests of the causal null hypothesis if the instrument used to proxy a drug target (i) is associated with the drug target (“relevance”); (ii) does not share a common cause with the outcome (“exchangeability”); and (iii) affects the outcome only through the drug target (“exclusion restriction”). Under the assumption of monotonicity, drug-target MR can provide valid point estimates for participants whose exposure is influenced by the instrument (i.e. a local average treatment effect).

Genetic instrument construction:

Genetic instruments for circulating APOC3, ANGPTL3, ANGPTL4, and PCSK9 concentrations were constructed from genome-wide significant (*P* < 5 x 10^-8^) and independent (LD r^2^ < 0.001) single-nucleotide polymorphisms (SNPs) in or within 1MB from the gene encoding the relevant protein using summary genetic association data from a prior GWAS in 35,559 individuals of Icelandic ancestry^6^. Replication analyses were performed in an independent GWAS of 54,219 participants of primarily white British ancestry in the UK Biobank. For SNPs that replicated (*P* < 0.05) and were directionally consistent, SNP weights were obtained from UK Biobank analyses. Such an approach mimics a “three-sample” MR design and has been shown to minimise bias from “Winner’s curse” in the presence of overlap of sample participants across “replication” and “outcome” data sources (i.e. UK Biobank participants in this analysis)^7^. Circulating APOC3 measures were not available in the UK Biobank and therefore no replication analyses were performed for this target. A genetic instrument for circulating CETP concentrations was constructed using genome-wide significant (*P* < 5 x 10^-8^) and independent (LD r^2^ < 0.001) SNPs associated with circulating CETP concentrations in or within 500KB from *CETP* in a GWAS of 5,706 participants in the Netherlands Epidemiology of Obesity (NEO) study^8^. In analyses exploring pathways mediating the effect of ANGPTL4, we also constructed a genetic instrument to proxy circulating triglyceride concentrations using genome-wide significant (*P* < 5 x 10^-8^) and independent (r^2^ < 0.001) SNPs associated with circulating triglycerides, irrespective of genomic position of variants, using data on ~1.3 million participants in the previously described GLGC analysis.

Colocalisation analysis:

Colocalisation analysis can permit evaluation of whether drug targets and disease endpoints are influenced by distinct causal variants that are in linkage disequilibrium (LD) with each other, indicative of horizontal pleiotropy (i.e. an instrument influencing an outcome through pathways independent to the exposure), a violation of the exclusion restriction assumption^9^. Colocalisation was performed by generating ± 100 kb windows around sentinel variants for drug targets using pair-wise conditional and colocalisation (PWCoCo) using default prior probabilities (*p1* = *p2* = 1 x 10^-4^, *p12* = 1 x 10^-5^).

**Observational analysis using directly measured pre-diagnostic circulating ANGPTL4 measures in EPIC**

Assessment of exposure:

Blood samples were collected at recruitment and underwent proteomic analysis by Somalogic using the SomaScan 7k Assay according to the manufacturers protocol. Briefly, the SomaScan platform uses modified nucleotides (Slow Off-rate Modified Aptamers; SOMAmer) which make direct contact with proteins, enabling detection of proteins or protein complexes and quantifies them in relative fluorescence units (RFUs) using DNA microarray^2^. Separate SOMAmers can bind to isoforms of the same protein but can also bind to the same protein at different sites (which can be impacted by post-translational modifications or complexes formed with other proteins). RFUs were first normalized by the manufacturer through the following steps: hybridization normalization, intraplate median normalization, plate scaling and calibration, and adaptive normalization to a population reference. We: i) log-transformed these normalized RFUs to reduce skewness; ii) excluded samples where the normalization scale factor was outside [0.4-2.5]; iii) excluded samples using principal component analyses (PCA) and a local outlier factor using a Tukey rule modified to account for skewness and multiple testing^10^; iv) applied plate correction using a residual approach whereby, for each SOMAmer, its measurements were corrected for plate effect estimated in linear mixed effect models adjusted for centre, age, sex, BMI, smoking status, and incidence of cancer, CVD, T2D and death, to preserve possible biological variation due to these factors^11^; v) centred and scaled measurements of each SOMAmer so that their mean and standard deviation in the sub-cohort were 0 and 1.

Multi-endpoint case-cohort design in EPIC:

A multi-endpoint case-cohort was established within EPIC in which individuals recruited in the UK, Netherlands, Spain, and Italy were eligible. Individuals were excluded if they had prevalent cancer or cardiovascular disease, had blood collection <35 or >75 years, or missing data for lifestyle or reproductive factors. Individuals were then included if they: were included in the EPIC-InterAct subcohort^12^, had a major cancer, or were a randomly selected type 2 diabetes, coronary heart disease, stroke, or death case. In the present study, we focus on individuals included in the EPIC-InterAct sub-cohort and/or the cancer case component which included a total of 10,261 individuals.

Cancer case definition in EPIC:

Incident first primary cancer cases were identified through a combination of centre-specific methods including health insurance records, cancer and pathology registries, and active follow-up through study participants and their next of kin. Follow-up for all individuals and events of interest began at recruitment and ended upon the occurrence of the event, loss to follow-up, or the last date of ascertainment, whichever came first. In the present study, cancer endpoints were defined as the first incident cancer diagnosis, using the following ICD-0-3 codes: colon: C180, C181, C182, C183, C184, C185, C186, C187, C188, C189; rectal (including rectosigmoid junction): C19.9, C20.9.

Covariate classification:

Age at event was defined as age at diagnosis or end of follow-up; 5-year age groups were defined from 0 to the maximum age of the included participants; BMI was measured as weight (kg) divided by height (m^2^); alcohol consumption was self-reported as grams/day; smoking was self-reported as any one of: never, former, current, unknown (never was used as the reference); physical activity was self-reported using the Cambridge physical activity index as any one of: inactive, moderately inactive, moderately active, active, missing (inactive was used as the reference; categorical); education level was self-reported as any one of: none, primary school completed, secondary school, technical/professional school, longer education (incl. University degree), not-specified (none was used as the reference); total daily energy intake was measured as kcal, total daily red meat intake was measured in grams, total daily processed meat intake was measured in grams, total daily fibre intake was measured in grams, and total daily calcium intake was measured in miligrams. Dietary variables were was collected at recruitment using validated country/centre-specific dietary questionnaires^13,14^. For individuals with missing dietary data, a missing category was used.

**Observational analysis using directly measured pre-diagnostic circulating ANGPTL4 measures in UK Biobank**

The design of the UK Biobank cohort study and the measurement of plasma proteins within the UK Biobank Pharma Proteomics Project have been reported previously^15,16^. In brief, the UK Biobank is a prospective cohort study of ~500,000 individuals aged 40–69 years when recruited in 2006–2010. Participants attended 1 of 22 assessment centres across England, Scotland and Wales where they completed a self-administered, touch-screen questionnaire and face-to-face interview, and trained staff took a series of measurements. 2,941 blood plasma analytes were measured across 54,219 UKB participants using the antibody-based Olink Explore 3072 proximity extension assay as part of the UK Biobank Pharma Proteomics Project (UKB-PPP).

Cancer case definition in UK Biobank:

Incident first primary cancer cases were identified through a combination of methods including GP records, health episode statistics (HES), and linkage to national cancer registries. Follow-up for all individuals and events of interest began at recruitment and ended upon the occurrence of the event, loss to follow-up, or the last date of ascertainment, whichever came first. In the present study, cancer endpoints were defined as the first incident cancer diagnosis, using the following ICD-0-3 codes: colon: C180, C181, C182, C183, C184, C185, C186, C187, C188, C189; rectal (including rectosigmoid junction): C19.9, C20.9.

Covariate classification:

Covariates classification in the UK Biobank analyses was the same as that employed in EPIC except for physical activity, alcohol consumption, red meat intake, processed meat intake, total daily energy intake, total daily calcium intake, and total daily fibre intake. Physical activity was self-reported using three MET (Metabolic Equivalent Task) scores based on IPAQ (International Physical Activity Questionnaire) guidelines: MET minutes per week walking, MET minutes per week moderate activity and MET minutes per week vigorous activity. Alcohol consumption was self-reported as any one of: daily or almost daily, three or four times a week, once or twice a week, one to three times a month, special occasions only, never, unknown (never was used as the reference). Red meat intake was self-reported using intake of beef, pork and lamb each as any one of: never, less than once a week, once a week, 2-4 times a week, 5-6 times a week, once or more daily, unknown (never was used as the reference). Processed meat intake was self-reported as any one of: never, less than once a week, once a week, 2-4 times a week, 5-6 times a week, once or more daily, unknown (never was used as the reference). Total daily energy, calcium and fibre intake were estimates calculated from self-reported 24-hour dietary recall questionnaires using only participants who self-reported that the last 24-hours was their typical dietary intake.

Multiple imputation:

To account for missingness in covariates included in Models 2 and 3, we performed multiple imputation using the Multivariate Imputation by Chained Equations (MICE) package in R. Multiple imputation was performed using predictive mean matching with 20 imputations and 10 iterations per imputation.

**Impact of ANGPTL4 loss-of-function on colon differential gene expression and gene set enrichment**

BarcUVa-Seq sample description:

Analyses were performed on 445 individuals (mean age 60 years, 64% female, 95% of European ancestry) who participated in a Spanish colorectal cancer risk screening program that obtained a normal colonscopy result (i.e., macroscopically normal colon tissue, with no malignant lesions).

RNA-Seq processing:

The raw RNA-Seq reads were processed using the BBTools suite to remove low-quality bases, adapters, and residual ribosomal RNA sequences^17^. The trimmed reads were then aligned to the human reference genome GRCh37 using STAR v2.5, with annotations from GENCODE release 19^18,19^. For further analysis, only samples with more than 10 million mapped paired-end reads, a unique mapping rate greater than 80%, and a multimapping rate lower than 15% were included. Gene expression was quantified using RSEM^20^.

Genotype processing:

Blood samples were genotyped using the Illumina OncoArray BeadChip^21^. The quality control criteria included the following: i) genotyping rate > 95%, ii) relatedness > 0.8, iii) SNP and per-sample missing rate > 0.1, iv) sex concordance between genetic data and reported information. Imputation was performed using the Haplotype Reference Consortium panel via the Michigan Imputation Server^22^. 6.9 million SNPs were retained after filtering for a minor allele frequency lower than 1% and an R 2 quality score greater than 0.7. SNP identifiers were annotated with dbSNP v142^23^. The concordance between genotype and RNA-seq samples was verified using CheckFingerprint^24^.

Transcriptome-wide gene expression and loss-of-function analyses:

Gene expression counts were normalised to account for library size differences using the trimmed mean of M-values (TMM) method^25^. Expression levels were inverse rank normal transformed. The models were adjusted for age, sex, sequencing batch, tissue location, the first two principal components of genetic ancestry, and 10 PEER factors^26^. eQTL identification was performed using linear models computed with FastQTL v211^27^. Effect estimates refer to the impact of the p.E40K minor allele (A) that causes genetic loss of ANGPTL4 function. A Benjamini-Hochberg FDR correction was used to account for multiple testing.

**Supplementary Materials Citations**

1 Michailidou, K. *et al.* Association analysis identifies 65 new breast cancer risk loci. *Nature* **551**, 92-94 (2017). <https://doi.org/10.1038/nature24284>

2 Fernandez-Rozadilla, C. *et al.* Deciphering colorectal cancer genetics through multi-omic analysis of 100,204 cases and 154,587 controls of European and east Asian ancestries. *Nat Genet* **55**, 89-99 (2023). <https://doi.org/10.1038/s41588-022-01222-9>

3 Schumacher, F. R. *et al.* Association analyses of more than 140,000 men identify 63 new prostate cancer susceptibility loci. *Nat Genet* **50**, 928-936 (2018). <https://doi.org/10.1038/s41588-018-0142-8>

4 Ebrahimi, E. *et al.* Cross-ancestral GWAS identifies 29 novel variants across Head and Neck Cancer subsites. *medRxiv*, 2024.2011.2018.24317473 (2024). <https://doi.org/10.1101/2024.11.18.24317473>

5 Phelan, C. M. *et al.* Identification of 12 new susceptibility loci for different histotypes of epithelial ovarian cancer. *Nat Genet* **49**, 680-691 (2017). <https://doi.org/10.1038/ng.3826>

6 Ferkingstad, E. *et al.* Large-scale integration of the plasma proteome with genetics and disease. *Nat Genet* **53**, 1712-1721 (2021). <https://doi.org/10.1038/s41588-021-00978-w>

7 Sadreev, I. I. *et al.* Navigating sample overlap, winner’s curse and weak instrument bias in Mendelian randomization studies using the UK Biobank. *medRxiv*, 2021.2006.2028.21259622 (2021). <https://doi.org/10.1101/2021.06.28.21259622>

8 Blauw, L. L. *et al.* CETP (Cholesteryl Ester Transfer Protein) Concentration: A Genome-Wide Association Study Followed by Mendelian Randomization on Coronary Artery Disease. *Circ Genom Precis Med* **11**, e002034 (2018). <https://doi.org/10.1161/circgen.117.002034>

9 Wallace, C. Statistical testing of shared genetic control for potentially related traits. *Genet Epidemiol* **37**, 802-813 (2013). <https://doi.org/10.1002/gepi.21765>

10 Breunig, M. M., Kriegel, H.-P., Ng, R. T. & Sander, J. in *Proceedings of the 2000 ACM SIGMOD international conference on Management of data* 93–104 (Association for Computing Machinery, Dallas, Texas, USA, 2000).

11 Viallon, V. *et al.* A New Pipeline for the Normalization and Pooling of Metabolomics Data. *Metabolites* **11** (2021). <https://doi.org/10.3390/metabo11090631>

12 Forouhi, N. G. & Wareham, N. J. The EPIC-InterAct Study: A Study of the Interplay between Genetic and Lifestyle Behavioral Factors on the Risk of Type 2 Diabetes in European Populations. *Curr Nutr Rep* **3**, 355-363 (2014). <https://doi.org/10.1007/s13668-014-0098-y>

13 Kaaks, R., Slimani, N. & Riboli, E. Pilot phase studies on the accuracy of dietary intake measurements in the EPIC project: overall evaluation of results. European Prospective Investigation into Cancer and Nutrition. *Int J Epidemiol* **26 Suppl 1**, S26-36 (1997). <https://doi.org/10.1093/ije/26.suppl_1.s26>

14 Riboli, E. & Kaaks, R. The EPIC Project: rationale and study design. European Prospective Investigation into Cancer and Nutrition. *International journal of epidemiology* **26**, S6 (1997).

15 Sudlow, C. *et al.* UK biobank: an open access resource for identifying the causes of a wide range of complex diseases of middle and old age. *PLoS Med* **12**, e1001779 (2015). <https://doi.org/10.1371/journal.pmed.1001779>

16 Sun, B. B. *et al.* Plasma proteomic associations with genetics and health in the UK Biobank. *Nature* **622**, 329-338 (2023). <https://doi.org/10.1038/s41586-023-06592-6>

17 *BBMap. SourceForge.*, <<https://sourceforge.net/projects/bbmap/>> (

18 Dobin, A. *et al.* STAR: ultrafast universal RNA-seq aligner. *Bioinformatics* **29**, 15-21 (2013). <https://doi.org/10.1093/bioinformatics/bts635>

19 Harrow, J. *et al.* GENCODE: the reference human genome annotation for The ENCODE Project. *Genome Res* **22**, 1760-1774 (2012). <https://doi.org/10.1101/gr.135350.111>

20 Li, B. & Dewey, C. N. RSEM: accurate transcript quantification from RNA-Seq data with or without a reference genome. *BMC Bioinformatics* **12**, 323 (2011). <https://doi.org/10.1186/1471-2105-12-323>

21 Amos, C. I. *et al.* The OncoArray Consortium: A Network for Understanding the Genetic Architecture of Common Cancers. *Cancer Epidemiol Biomarkers Prev* **26**, 126-135 (2017). <https://doi.org/10.1158/1055-9965.Epi-16-0106>

22 Das, S. *et al.* Next-generation genotype imputation service and methods. *Nat Genet* **48**, 1284-1287 (2016). <https://doi.org/10.1038/ng.3656>

23 Database resources of the National Center for Biotechnology Information. *Nucleic Acids Res* **44**, D7-19 (2016). <https://doi.org/10.1093/nar/gkv1290>

24 *picard/src/main/java/picard/fingerprint/CheckFingerprint.java at master · broadinstitute/picard. GitHub*, <<https://github.com/broadinstitute/picard/blob/master/src/main/java/picard/fingerprint/CheckFingerprint.java>> (

25 Robinson, M. D. & Oshlack, A. A scaling normalization method for differential expression analysis of RNA-seq data. *Genome Biol* **11**, R25 (2010). <https://doi.org/10.1186/gb-2010-11-3-r25>

26 Stegle, O., Parts, L., Piipari, M., Winn, J. & Durbin, R. Using probabilistic estimation of expression residuals (PEER) to obtain increased power and interpretability of gene expression analyses. *Nat Protoc* **7**, 500-507 (2012). <https://doi.org/10.1038/nprot.2011.457>

27 Ongen, H., Buil, A., Brown, A. A., Dermitzakis, E. T. & Delaneau, O. Fast and efficient QTL mapper for thousands of molecular phenotypes. *Bioinformatics* **32**, 1479-1485 (2016). <https://doi.org/10.1093/bioinformatics/btv722>
